# A comprehensive review of biological and genetic control approaches for leishmaniasis vector sand flies; emphasis towards promoting tools for integrated vector management

**DOI:** 10.1371/journal.pntd.0012795

**Published:** 2025-01-27

**Authors:** Yasoda Kumari, Nayana Gunathilaka, Deepika Amarasinghe

**Affiliations:** 1 Department of Parasitology, Faculty of Medicine, University of Kelaniya, Ragama, Sri Lanka; 2 Department of Zoology and Environment Management, Faculty of Science, University of Kelaniya, Dalugama, Sri Lanka; University of Lucknow, INDIA

## Abstract

**Background:**

Leishmaniasis is a health problem in many regions with poor health and poor life resources. According to the World Health Organization (WHO), an estimated 700,000–1 million new cases arise annually. Effective control of sand fly vector populations is crucial for reducing the transmission of this disease. Therefore, this review aims to comprehensively examine and evaluate the current methods for controlling sand fly populations, focusing on biological and gene drive techniques.

**Methods and findings:**

A detailed, comprehensive literature search was carried out using databases including Google Scholar, PubMed, ScienceDirect, and the National Library of Medicine (NIH). These searches were done using specific keywords related to the field of study. This current review identified several promising methods, including genetically modified sand flies, using transgenic approaches by taking advanced gene editing tools like Clustered Regularly Interspaced Short Palindromic Repeats (CRISPR/Cas9) and genetic modification of symbiotic microorganisms for controlling sand fly populations, which appeared to be proven under laboratory and field settings.

**Conclusion:**

Genetic control approaches have many benefits over chemical control, including long-lasting effects on targets, high specificity, and less environmental impact. Advances in genetic engineering technologies, particularly CRISPR/Cas9, sterile insect techniques, and gene drive insect modification, offer new avenues for precise and efficient sand fly management. Future research should prioritize optimizing rearing and sterilization techniques, conducting controlled field trials, and fostering collaboration across disciplines to realize the potential of genetic control strategies in combating leishmaniasis.

## Author summary

This review explores new ways to control sand fly populations responsible for spreading leishmaniasis, a disease affecting millions worldwide. Traditional methods, such as insecticides, are not always effective and can harm the environment. We look at genetic techniques such as the sterile insect techniques, which involves releasing sterilized male sand flies to reduce the population, and other methods like gene drives and paratransgenesis, which use genetic modifications to control sand flies. Our review highlights the need for further studies to optimize these techniques and understand their impacts better. By advancing genetic control methods, we aim to find more sustainable and effective ways to combat leishmaniasis and improve public health.

## Introduction

Leishmaniasis is a health issue in many regions with poor health and life resources. According to the WHO, an estimated 700,000–1 million new cases arise annually [1]. The disease leishmaniasis is caused by over 20 species of protozoan parasite, *Leishmania*, and transmitted by insect vector Phlebotomine sand flies (Diptera, Psychodidae) [2]. Of about 1000 reported sand fly species, 98 have been confirmed and/or suspected as vectors of important medical diseases, including Chandipura encephalitis, vesicular stomatitis and leishmaniasis being the most important [3]. The severity of leishmaniasis exhibits three clinical forms: cutaneous leishmaniasis, mucocutaneous leishmaniasis, and the most fatal visceral leishmaniasis (VL) [1].

Although early diagnosis and treatments are crucial in controlling the disease [4], there is a need for proper systemic disease surveillance, appropriate diagnostic tools and expertise to carry out control measures. Human vaccines against *Leishmania* parasites are still under trial [5,6]. Consequently, the most effective way of this control is avoiding vector bites and implementing suitable vector control strategies [7].

Vector control strategies that WHO recommended are not fully effective in controlling transmission of this disease since the sole application of insecticide against adult sand flies is unsuccessful due to resistant development, as well as recommended personal protective measures, including the use of insecticide-treated bed nets, clothes and use of insect repellent seems to be unsuccessful in some conditions and the best point is these control measures are often not accessible for people in low-/middle-income population where disease burden is already exist [8,9]. There is a need to find a cost-effective and productive control measure to apply under field conditions against vector sand flies, as leishmaniasis is a considerable social and economic burden in some parts of the world.

Therefore, researchers and health professionals exploring methods to control insect vectors using concepts called biocontrol and gene drive techniques that involve the utilization of natural parasites, predators, entomopathogens, and genetically modified vectors as an alternative strategy that could be incorporated into integrated vector management (IVM). These approaches are gaining much interest as environment-friendly vector control strategies [10] because these methods are more advantageous through having less harmful effects on humans and other beneficial insects than synthesized chemicals. On that account, this comprehensive review aims to summarize various biological and genetic approaches that have been given proof of success under laboratory and field conditions against leishmaniasis vector sand flies and to explore how these methods could be applied to IVM strategy as an alternative sustainable approach to mitigate leishmaniasis transmission.

## Methodology

This is a narrative review of the vector control tools used, tested, and evaluated in controlling the leishmaniasis vector sand flies. A detailed literature search was conducted using databases including Google Scholar, PubMed, ScienceDirect, and the National Library of Medicine (NIH). The primary keywords used in the search were “leishmaniasis vectors,” Sand flies,” “biological control,” “genetic control,” “vector control,” “Paratransgenic,” “predators and parasites,” and some other linked search terms for papers published in the English language up to April 2024. The collected data were correctly cited. The articles that were duplicated or not relevant directly to the proposed objectives were excluded. Based on the literature review, chemical control, biological control approaches, genetic/vector modification approaches, and paratransgenic aspects were included in this review. The search was limited to articles published in English.

**Fig 1:**
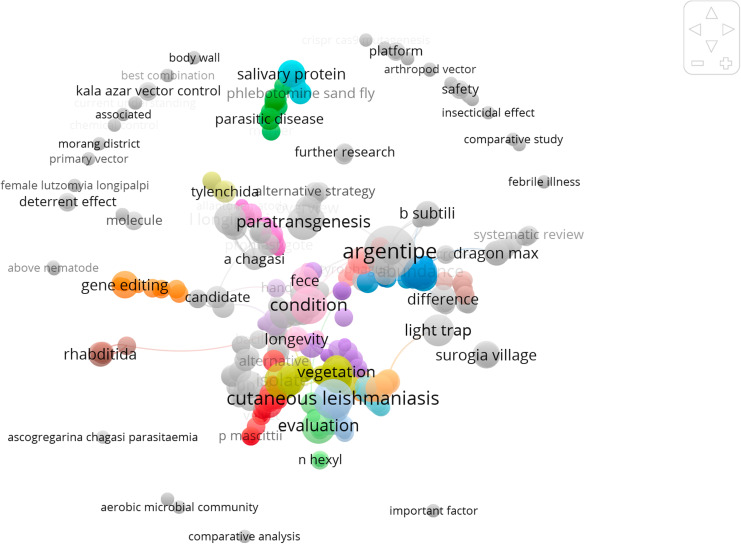
Network mapping of co-occurrences of keywords.

A bibliographic analysis was performed to identify the current research status and future potential relationships with the terms linked to the control of sand flies. The VOSviewer (version 1.6.20) software was used to construct and map the data (Fig 1).

## Results

### Chemical control of sand flies

Controlling sand fly populations is crucial for reducing leishmaniasis transmission. Chemical interventions, such as indoor residual spraying (IRS), insecticide-treated nets (ITNs), space sprays (fogging), chemical repellents such as dimethyl phthalate, and pyrethrum and the use of impregnated dog collars (IDCs) proved to be efficient in sand fly control through sand fly density reduction or preventing vector–host interactions [11–13].

Control of vectors could be achieved either by interrupting female vector–host interaction or through vector reduction approaches [14]. In the era of the malaria burden, dichlorodiphenyltrichloroethane-based IRS was employed for sand fly control and malaria elimination plans in several countries, including Syria, Nepal, Iran, and India [5,11]. Currently, synthetic chemicals have been used in a broad array of different modes to control vector sand flies in leishmaniasis endemic areas.

#### Indoor residual spraying (IRS).

The IRS application for household walls using residual insecticides (pyrethroid, organophosphate, carbamate, and organochlorine) is considered the primary method of chemical control in high-disease-burden localities [5,13]. Pyrethroids are the leading insecticide group the IRS uses globally [5]. It has proven promising results in reducing sand fly population over several geographical areas, including Bangladesh [15], India [16,17], and Nepal [18]. The choice of insecticides for IRS determines the success of those interventions against vector control. Several insecticides and combinations of chemicals have proved to be effective in suppressing different sand fly populations over many areas, including deltamethrin (over 90% suppression of vectors) [13,19], and breeding sites treated with the combination of deltamethrin with tempos have given positive outcomes [13]. However, some studies conducted in Bangladesh have reported reduced prolonged effects of the sole application of deltamethrin [20]. Using alpha-cypermethrin [16,18,21] and lambda-cyhalothrin [17] has yielded mixed results across different studies.

With new techniques and facilities available, novel approaches have been introduced to apply chemical interventions. Insecticide-treated durable wall lining (DWL) is an alternative to IRS. This method utilizes a thin insecticide-treated polyethylene net that covers the inner wall surface [22]. This novel technique has been effective in reducing the local sand fly population in some countries [13,23]. However, high cost and disposal/handling issues with large volumes of these chemical-treated plastics have led to the discontinuation of DWL application [5].

#### Insecticide-treated nets (ITNs).

Insecticide-treated nets (ITNs) and long-lasting ITNs (LLINs) hold significant vector control tools utilizing safe chemicals for humans, such as pyrethroid and deltamethrin. The ITNs combine physical and chemical actions against vectors and are cost-effective compared to other chemical-based methods. Although the use of ITNs has gained varying prominent results [9,24], in certain instances, these approaches weren’t effective due to several factors, including logistic constraints, short residual efficacy, lack of community participation, and the development of insects’ resistance against applied chemicals [25].

Field trials and studies emphasize varying degrees of effectiveness in applying ITNs and LLINs. A Bangladesh study [15] demonstrated the applicability of KOTAB-impregnated bed nets and LLINs where LLINs showed a prolonged effect in reducing vector density ranging from 9%–78% over nearly 2 years and KOTAB was productive only up to a certain follow-up point. This study also evaluated the combined control measures and has given promising results in sand fly population over time, ranging from 16% to 86% for the combined interventions of IRS + LLIN and IRS + KOTAB and 26% to 86% for LLIN and Outdoor sprays [15]. Community-wide trials that were carried out in Sudan and the Middle East have found that ITNs were effective in reducing leishmaniasis burden by 59%–98% for at least 1 year [5]. Upon employing this strategy for leishmaniasis control, many geographics, including Iran [26], Turkey [9], and Bangladesh [15] have shown satisfactory results over many years, from 1–3 years.

#### Space spraying/fogging.

Even though the use of fogging method of insecticide application seems to be somewhat effective in case of mosquito control [27], there is only limited evidence available on the success of this method against the control of sand flies [12,28] due to its scant habitat coverage and limited residual activity [5,12]. Space sprays, including cold fogging, thermal fogging, and ultra-low volume applications, have been studied in various ecological settings and have shown mixed results of these control approaches on sand flies. The diffusion property of thermal fogging makes it an effective method for targeting adult sand flies that breed and rest in caves, crevices, and spaces between rock piles, and thermal fogging has proven control efficiency on sand flies of several species, including *Phlebotomus* spp. [29], *Lutzomyia gomezr*, *Lu. Panamensis*, *Lu. Dyponeta*, and *Lu. Triramula* [30].

#### Chemical-based personal protection and repellents.

Natural or synthetic compounds that have repellent properties against insects are applied at the household level or directly on the body/clothes as a personal protection method [5]. These compounds are commercially/naturally available in various forms, such as insecticide-treated cloths, topical repellents for direct application on skin, and spatial repellents that release human-friendly chemicals into the air, such as coils, candles, and vaporizers. Topical repellents like diethyl-m-tolumide (DEET), ethyl butylacetylaminoproprionate (IR3535), SS220, picardin, ethoxy-diethylbenzamide, and chloro-diethylbenzamide [31] directly apply to the skin and it act as protective barrier against several insect bites including sand flies. A study by Naucke and colleagues [32] evaluated the efficacy of 10% IR3535 as a repellent against two species of sand flies and found that the mean protection time was 5.9 h for *P. duboscqi* and 10.4 h for *P. mascittii,* indicating a level of repellency varies by species. A study that evaluated IR3535 on *P. papatasi* found that the complete protection time was more than 6 h and also observed that IR3535 has a limited spatial repellent efficacy as sand flies attempted to land, but frequently veered away from the skin [31]. A comprehensive review by Amane and colleagues [33] has documented the plants/plant-derived products that can be used as repellent agents against sand flies. The findings of this review conclude that there are many medicinal plants/extracts, including neem oil, *Monticalia greenmaniana* (Asteraceae), *Nicotiana tabacum* (Solanaceae), *Cymbopogon citratus*, and *Eucalyptus staigeriana* that have repellent properties against several insects including sand flies.

#### Impregnated dog collars (IDCs).

IDCs are mainly used in regions where reservoirs play an essential role in leishmaniasis transmission. Usually, these collars are treated with deltamethrin to provide a barrier that repels sand fly contact with these canine reservoirs [34]. In various regions, these insecticide-treated dog collars have been extensively tested and proven to effectively reduce infection risk in dogs [34,35]. A study by Courtenay and colleagues [36] evaluated the effectiveness of these dog collars through a pair-matched–cluster randomized controlled trial and found that these collars provide 50% (0·95/1000/year compared to 1·75/1000/year) protection against infantile VL incidences indicating the significance of applying this control approach for IVM programs.

### Chemical-based control of immature stages of sand flies

The primary purpose of chemical control of sand fly immatures is to prevent them from developing into adults. Still, due to difficulties in finding exact breeding grounds [36], only limited evidence is available on insecticide application to sand fly larval stages. Several studies have evidenced effective chemicals with larvicidal activity against sand fly immatures under laboratory and field conditions. Larvicidal effect of pyriproxyfen on *Lu. longipalpis* interrupt the pupal stage developments, and thus prevent adult emergence [37]. A study by Gómez-Bravo and colleagues [38] investigated the combined chemical formulation for controlling *Lu. longipalpis* was done through larval control, and they used commercial insecticide formulations, one with permethrin and pyriproxyfen as active ingredients and the other with only permethrin. Applying these insecticides targeted at chicken coop grounds and the Dragon Max formulation led to a significant reduction in vector population for an extended period of 21 weeks, indicating the importance of combined active ingredients formulation as larvicides for sand fly immatures.

Although chemical control is efficient in controlling and eliminating sand flies, there are many drawbacks related to chemicals and their application in the field. Inappropriate use of chemicals leads to insecticide resistance [39] and causes negative impacts on other non-harming insects, humans, and the environment. Side effects arise due to the use of currently available treatments and the unavailability of successful vaccines for disease control, which are major constraints in managing leishmaniasis [6]. Therefore, eliminating leishmaniasis mainly relies on targeting control of vector sand flies using commercially produced insecticides and proper environmental management strategies [11]. As detecting the exact habitats of sand fly larvae is quite difficult, vector control approaches only target sand fly adults. Therefore, there is a need for alternative vector control methods that could be applied to be more effective in controlling sand fly vectors and ultimately minimize adverse impacts on others. Hence, through this review, we focus on advancing emerging novel sand fly targeted vector control strategies, including paratransgenesis and other biological methods in preventing leishmaniasis.

### Biological control approaches for sand flies

Chemical control, primarily through insecticides, has been a mainstay since World War II. However, insecticide resistance and environmental impact necessitate exploring alternative methods, including biocontrol approaches that use natural organisms to control sand fly populations. Using biological control measures like natural predators and pathogens provides sustainable alternatives to chemical-based insecticides for sand fly control. The application of bioagents or measures is more advantageous through efficiency in reducing the sand fly population, providing user-friendly ecological sustainability, and ensuring public health and safety. Parasites like fungi, mites, and nematodes act as endo- and/or ectoparasites of sand flies and some of the parasites have lethal effects on sand flies [40]. Sand flies prefer to rest in habitats with dark and damp places that provide suitable climate factors for their survival, like animal sheds, tree holes, caves, and cracks and crevices. The prevailing characteristics in these habitats also provide flavor for other organisms that could act as predators/entomopathogens of these sand flies, suggesting the suitability of biological agents to control these vectors [40]. The critical biological-based control approaches that have been tested are summarized below.

#### Entomopathogens.

The organisms that can cause harmful effects, such as diseases and/or death of insects, are categorized as entomopathogens. So far, from various findings, several species of entomopathogens have been discovered belonging to bacteria, fungi, viruses, protozoa, mites, and nematodes that have mild-to-lethal effects using various aspects like pressure on sand fly survival, biology, fertility, and by affecting their reproductive capacity [10].

#### Entomopathogenic nematodes.

Entomopathogenic nematodes (EPNs) are emerging as an effective alternative to control insect vectors. These EPNs are naturally found in soils that can attack various hosts, including sand flies [41]. The mode of action of these EPNs relies on their relationship with symbiotic bacteria. Once they attach or infect a host, they release their symbiotic bacteria, and as a combination effect of these bacterial and nematode actions, the host dies within a short period [10,42]. The use of EPNs from the Steinernematidae and Heterorhabditidae families [10] is considered as highly effective as biocontrol agents as the rapid mortality (within 24–48 h) induced by nematodes belonging to these families is facilitated by their symbionts (*Xenorhabdus* spp. and *Photorhabdus* spp.) [10]. The notable success of various nematode species in targeting different sand fly vectors, such as *Tricephalobus steineri* and *Procephalobus* sp., effective in reducing *P. papatasi* populations [43], *Anandranema phlebotophaga* caused infertility in *Lu. longipalpis* [44] and in *Lu. fischeri* [10], *Didilia ooglypta* affected the development and longevity of *P. papatasi* and *P. sergenti* [45]. Nematodes from the Steinernematidae family showed a 42%–94% mortality rate in *P. papatasi* larvae [46].

A laboratory sand fly colony of *P. papatasi*, which was studied by Temeyer and collogues [43], suddenly crashed, and they found the nematode *T. steineri*/*Procephalobus* sp. infection as the cause for the loss of sand fly colony productivity.

#### Entomopathogenic protists.

Entomopathogenic protists also have a significant effect on controlling sand flies, and in the case of *Leishmania* vector, the only studied protists are gregarines. Slavica Vaselek [10] has well reviewed the potential use of these protists against sand fly control. Gregarines are the most studied entomopathogenic protists in sand flies. Species such as *Psychodiella chagasi*, *Ps. mackiei*, *Ps. saraviae*, *Ps. sergenti*, and *Ps. tobbi* infect sand flies by attaching to the host’s digestive tract or body cavity and completing their life cycle within the host [10]. *Ps. chagasi* significantly reduces the longevity of *Lu. longipalpis* adults, found in multiple sand fly species [47]. *Ps. sergenti* affects the survival of *Phlebotomus sergenti* and *Ps. tobbi* negatively impacts their host’s longevity [48]. Gregarine infections reduce the resistance, development, and reproduction of sand flies, suggesting a potential for their use in biocontrol, although their study is still rudimentary.

#### Mites.

Mites have proved as one of the most significant groups that could be parasitic and/or phoretic for sand fly larvae and adult populations, affecting sand flies by attaching to their exoskeleton and feeding on them [10]. Some mite species can parasitize and infect sand fly larvae, pupae, and adults by attaching to sand flies’ bodies and obtaining nutrition.

Through different studies and field evaluations, 15 mite families associated with sand flies have been identified so far [49]. Among these 15, few consider an essential group of terrestrial parasite genes including Microtrombidiidae, Erythraeidae, and Trombidiidae, which have the ability to act as ectoparasites during sand fly’s different life stages of larval, nymph, and adult stages [10].

These mites have been identified for their exclusive behavior for reducing sand fly larvae, eggs, or adult populations, and this phenomenon has been observed through different laboratory-based studies. Dinesh and colleagues [40] investigated the possibility of using biological control agents for sand fly control. They demonstrated significant effects of mites on both sand fly larvae and adults. They concluded that the use of mites against sand fly control could be more advantageous rather than the use of chemical insecticides. Specific mite species and their sand fly hosts include: *Biskratrombium coineaui* parasitizes *P. papatasi* adults [50] and *Microtrombidium hindustanicum* is associated with *P. papatasi*, *P. argentipes*, and *P. sergenti* [51]. Mites from the Stigmaeidae family, particularly species from the genus *Eustigmaeus*, have been frequently found parasitizing sand flies and include *E. dyemkoumai* (parasite of *Phlebotomus duboscqi*) and *E. gamma* (parasite of *Phlebotomus pius*) [52].

#### Spiders.

As a natural predator with a broad diet preference, including insects, spiders have a significant impact as biocontrol agents for leishmaniasis vector control. The capability of capturing and ingesting many preys within a short time confirms their usability as a natural bio-control agent for sand fly control where applicable. Spiders are most efficient in predating adult sand flies [40]. This is likely to be true in the case of natural habitats since spiders inhabit different environmental settings like grasslands, forests, and other natural and artificial habitat dwellings. The distribution of spiders in these habitat types ensures the possibility of being a predator that can capture various insect species, including sand fly adults. Observing the predatory action of an unidentified spider species that attacked blood-fed sand flies gives a positive insight into the potential utilization of spiders in the field of biocontrol [40]. The predatory mechanism observed through that study highlights the step-by-step consumption of attacked sand fly, which contributes to the reduction of these sand fly vectors, and it is important to focus on the fact that spider behavior was explicitly aimed at the blood-fed sand fly. This proves the significance of spider predators in reducing the reproductive potential of female sand flies since only females take blood from their respective hosts.

### Use of Bti bacteria as a biological larvicide

Bacteria as entomopathogens can be linked in different ways, such as pathogens, larvicides, and paratransgenic tools to control vector sand flies [10]. Bacteria can infect and kill their host sand fly, reducing the host population. In view of sand flies, several studies [53,54] have focused on these bacterial effects on sand flies’ different life stages. Bacterial species like *Bacillus thuringiensis israelensis* (Bti) that have parasitic ability against sand flies have been tested and results imply a significant effect on both sand fly larvae and adults [5,12].

#### Fungi.

Among the available biocontrol agents for sand fly control, fungi stand out as one of the significant categories that can control sand flies [55]. These fungi exert their pathogenic behavior on insect vectors through a sequence of actions where they can have a lethal effect on these insects. Different species of entomopathogenic fungi (EPF) may exhibit variability in their action and the level of lethal effect on a particular insect. The degree of attachment and the penetration ability determine their suitability as an EPF and the scope of its impact on the insect [56]. A comprehensive review by Singh, Raina, and Singh [56] on the detailed mechanism and steps involved in the virulence mechanism has been well documented. Studies show these EPFs are efficient in controlling sand flies regarding the alternative strategy for leishmaniasis control. Kasili and colleagues [57] carried out a pilot study to find out the EPF potential of one of the fungal species that inhabit soil namely *Metarhizium anisopliae* against sand flies and found that this fungal isotope as one of the feasible candidates to develop sand fly control plans under field conditions. *Beauveria bassiana* is one of the well-studied EPFs proven to be efficient as a biocontrol agent against several insect vectors like *Aedes aegypti* [58], and sand flies [59–61]. A recent study by Pirmohammadi and colleagues [59] evaluated the *B. bassiana* fungal effect on different life stages of *P. papatasi* and the findings of this study concluded that these fungal isolates have considerable biocontrol capacity against adult sand flies.

Apart from mainly using *B. bassiana*, other fungi species, namely *M. anisopliae* [57,61,62], have been documented as biocontrol agents against sand flies. These two fungi species are currently used as EPF for trial studies for sand flies. Two studies have shown contrasting results on the pathogenicity of these two fungal species against sand flies. Ahmed and colleagues [62] compared the effect of these two fungi species *B. bassiana* and *M. anisopliae* effect on different growth stages of sand fly *P. papatasi,* and the findings revealed that *B. bassiana* was effective in fast adhesion, germination, and penetration into sand flies’ body. Ngumbi and colleagues [61] observed contrast results by a laboratory study to evaluate the pathogenicity of *M. anisopliae* and *B. bassiana* against *P. duboscqi*, where they observed the pathogenic effect of *M. anisopliae* was better than that of *B. bassiana* in vitro conditions where results have shown that 100% mortalities due to *M. anisopliae* infection. The variability of these results might be due to the experimental setup, the study of fungal strains and sand fly species, and environmental factors.

Although EPF against sand fly control has proven as one of the promising biocontrol agents, an effective sand fly targeted delivery mechanism is crucial when applying this method in practice. Otherwise, when conducting field-based trials or applications, the potential of these agents against sand flies might be misleading. It may cause several failures, such as inadequate contact of fungal strains with target insects, which could reduce fungal infections on vectors. An earlier study by Warburg [60] proposed that the use of fungal spore-smeared papers to hang in darker places where sand flies prefer to rest would be a better approach to deliver EPF efficiently as these spores remain considerably more extended period to infect sand flies. Spore sprays are also a better approach to applying EPF to less accessible places, as proposed by Warburg [60]. An overview of entomopathogens that have potential use in sand fly control is summarized in Table 1.

**Table 1:** Overview of entomopathogens.

Entomopathogen	Mechanism of action	Entomopathogen sp. and target sand fly species	References
Entomopathogenic nematodes (EPNs)	Symbiotic relationship with bacteria: infects host, releases bacteria, leading to host death within 24–48 h. Highly effective against various sand fly vectors, inducing rapid mortality rates.	*Tricephalobus steineri* and *Procephalobus* sp. effective against *P. papatasi* *Anandranema phlebotophaga* effective against *L. longipalpis* and *L. fischeri* *Didilia ooglypta* against *P. papatasi* and *P. sergenti* *T. steineri*/*Procephalobus* sp. against *P. papatasi*	[10,43–45]
Entomopathogenic protists	Attach to the host’s digestive tract or body cavity, completing their life cycle within the host. Reduce development, and reproduction of sand flies.	*Psychodiella chagasi*, *Ps. mackiei*, *Ps. saraviae*, *Ps. sergenti*, and *Ps. tobbi* *Ps. chagasi* against *Lutzomyia longipalpis* adults *Ps. sergenti* against *P. sergenti*	[10,47,48]
Mites	Parasitize and/or feed on sand fly larvae and adults, affecting their health and longevity.	Mite families: Microtrombidiidae, Erythraeidae, and Trombidiidae as ectoparasites *Biskratrombium coineaui* against *P. papatasi* *Microtrombidium hindustanicum* against *P. papatasi*, *P. argentipes*, and *P. sergenti*	[10,50,51]
Spiders	Predatory behavior capturing and ingesting sand flies. Effective in reducing the reproductive potential of female sand flies.	Unidentified sp.—effective against blood-fed female *P. argentipes* sand flies.	[40]
Bacteria	Infect and kill sand flies, acting as a biological larvicide. Significant effects on both larvae and adult sand flies.	*Bacillus thuringiensis israelensis* (Bti) against several sand fly sp. including *P. papatasi*, *P. argentipes* and *Lu. longipalpis*	[5,12]
Entomopathogenic fungi	These fungi exert their pathogenic behavior on insect vectors through a sequence of actions where they can have a lethal effect on these insects. Pathogenic behavior includes attachment, germination, and penetration into the host.	*Beauveria bassiana* against *papatasi* and *Metarhizium anisopliae* against *duboscqi*	[59,61]

### Modification of vectors

Although various above-reviewed sand fly control measures exist, genetic control has received more attention and interest worldwide with technological advancement. The potential application of genetic control measures could be linked with the current use of gene drive techniques for other insects as these approaches are not well confirmed in sand flies. Undiscovered control approaches for sand flies like endosymbiotic bacterium *Wolbachia* that is successfully proven and employed as a biocontrol candidate for mosquitoes due to its ability to suppress mosquito populations in different ways [63,64]. The concept of the genetic modification of sand flies to produce sterile male insects to suppress vector populations, as well as the utilization of transgenic approaches by taking advanced gene editing tools like CRISPR/Cas9, could also be taken into account for future aspects in concerning sand flies [65,66].

The aforementioned genetically modified techniques are brought into work by injecting genetic constructs into the insect’s early life stages, typically using the insect’s eggs. Jeffries and team [65] studied a novel protocol to obtain and microinject eggs of *Lu. longipalpis* utilizing a strain of *Wolbachia* as a marker. This study succeeds in producing early-generation *Wolbachia* transinfected *Lu. longipalpis* lines indicate this technique as a potential strategy for future perspectives.

**Fig 2:**
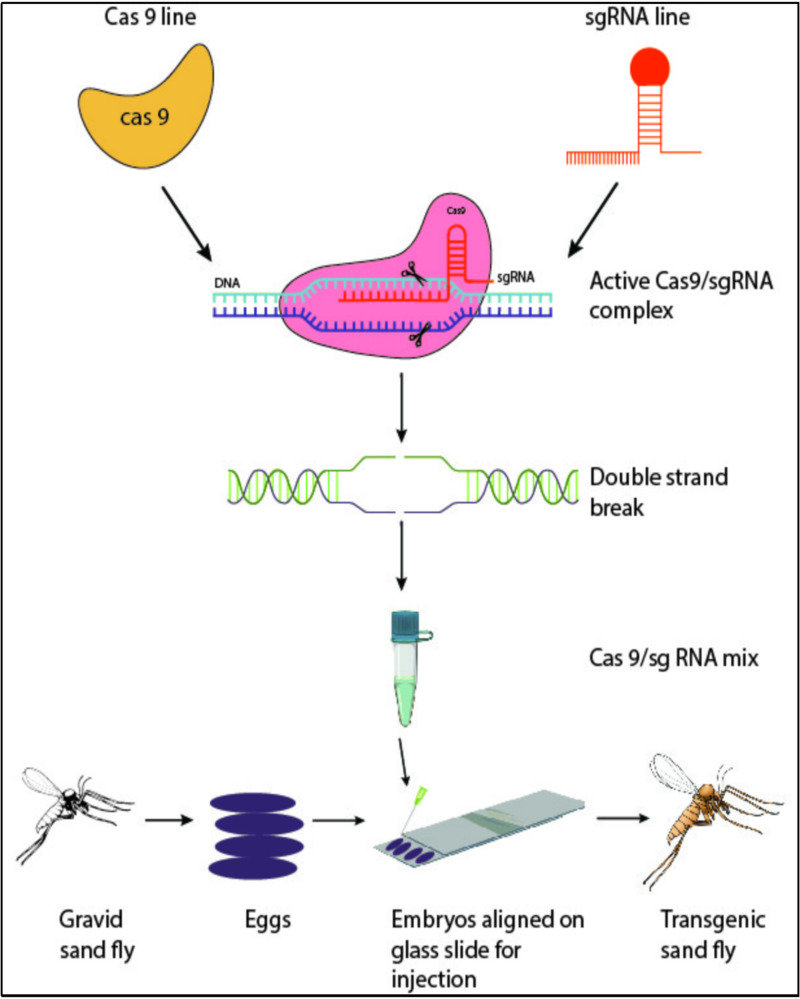
Schematic representation of CRISPR/Cas9 in sand fly vector research; the CRISPR/Cas 9 system comprises sgRNA and Cas9. The main steps include selecting the target gene, sgRNA design & production, Cas9 production, and CRISPR/Cas9 mix microinjection into sand fly embryo.

CRISPR/Cas9 is one of the excellent genomes editing tools that make it possible to produce genetically modified organisms (GMOs) by altering the DNA of an organism. This has given new avenues to control disease-transmitting vectors. This CRISPR/Cas9 system has been well-studied for several vector insects, including *Anopheles* sp. [67] and *Aedes* sp. [68]. Since there are no reported attempts to apply this technique for sand flies, Martín and colleagues [69] documented a detailed protocol to achieve sand fly microinjection, which is one of the essential steps in utilizing the CRISPR/Cas9 system for sand fly control (Fig 2). This study also described several issues related to gene editing and microinjection concerning non-model organisms like sand flies, and those challenges could be carefully addressed for future laboratory studies when developing a successful strategy.

As an initial move towards using the CRISPR/Cas9 technique concerning control of *Leishmania* transmission, Louradour and team [70] showed the first successful in vitro mutagenesis of vector *P. papatasi* and *Lu. longipalpis*. This trial study was able to establish an early-generation mutant that expressed increased susceptibility to *Leishmania major* infection under in vitro conditions as they found that the survival rate of injected embryos was low (11 out of 540), the efficiency of mutagenesis was high with 8 out of 11 surviving adults. Additionally, *Wolbachia* strains have been naturally found in *Phlebotomus* and *Lutzomyia* species, suggesting the potential for using *Wolbachia* alone or combined with the sterile insect technique (SIT) [71].

### Paratransgenic techniques for sand fly control

Paratransgenesis is a novel application used to control several vector-borne diseases, including dengue [72] and many other diseases. The concept of this technique lies within the genetic modification of symbiotic microorganisms to express genes that could interfere with the pathogens transmitted by that insect species [73]. The general process of paratransgenesis involves the isolation of native symbionts or commensals from vector species and then genetic modification is done to bring out anti-pathogenic symbionts under laboratory conditions. These modified microorganisms are then reintroduced into the vector insect, where these altered symbionts can interrupt the pathogen’s life cycle within the insect host [10]. The microorganisms most commonly used for this technique are symbiotic and/or commensal bacteria, which have been proven to affect expressing anti-parasitic behaviors once they are genetically manipulated. Bacterial symbionts, fungi species, and occasionally viruses [73] are also utilized for this purpose, where technological advancement changes the medical sector to facilitate a better human life.

Successful application of paratransgenic technique in controlling vector-borne pathogens relies on several requirements. In summary, there should be availability of culturable microorganisms under in vitro conditions, symbionts should be able to colonize in all development stages of the insect from instars to adults, modified microbes required to be safe and non-pathogenic to other animals and humans, modified microbes should able to maintain its fitness and stability with insects body. They must not be compromised, and it is essential to have an efficient mechanism to introduce and disperse the modified symbionts into the vector insect under practical situations. A comprehensive review of these requirements has been published by several authors in their review articles [7,73], highlighting the significance of this advanced technique in controlling pathogens transmitted by insect vectors.

Many researchers [74–76] have studied suitable bacterial species that could be used to achieve paratransgenic techniques related to leishmaniasis transmission by sand flies. An adult sand fly may acquire bacteria from external sources in three ways. During feeding sugar sap, from the host’s skin when it takes a blood meal and through transstadial transmission from larvae to adults [77]. Understanding these acquisition routes is essential for applying paratransgenic techniques against parasites [78]. One of the challenges associated with this paratransgenesis technique is finding a robust successful delivery system to expose modified symbionts into the sand fly vectors [78]. Therefore, research studies focus on discovering efficient delivery tools to ensure the optimal establishment of this technique under field conditions.

A research team [79] discovered an exciting point highlighting the application of non-harmful soil microbes as a delivery vehicle for anti-pathogenic compounds to control *Leishmania* transmission by Phlebotomine sand flies. Symbiotic or commensal microbe composition within an insect sand is always associated with the environment in which they reside [75]. A study by Hurwitz and team [80] demonstrated the feasibility of a paratransgenic approach using symbiotic *Bacillus* spp. under laboratory conditions using *P. argentipes*, the primary vector species of VL in India. In this study, modified *Bacillus subtilis* was introduced into a sterilized larval diet, and the microbial load was evaluated during different development stages of sand fly to identify environmental bacteria as a tool for sand fly control. Results of this study highlight that this genetically modified bacterium has a disruptive effect on *Leishmania donovani* development within sand fly midgut [80].

A study by Ghassemi and colleagues [78] tested another delivery method using rodent food baits that contain symbionts to evaluate the efficacy of this delivering mechanism under both laboratory and field conditions. In this study, two bacterial species, namely *Serratia* AS1 and *Enterobacter cloacae*, were introduced into rodent food bait, and it was provided to a rodent species, *Rhombomys opimus*. These modified bacteria are then released to sand fly’s breeding grounds through rodents’ fecal matter. The findings of this study proved that *Serratia* AS1 bacteria can successfully be introduced into adults through transstadial transmission, where rodents act as carriers for genetically modified bacterium, but *E. cloacae* wasn’t able to transmit using this route. Based on results obtained by Hillesland and team [75], they indicated that two non-pathogenic bacterial species, namely *Bacillus megaterium* and *Brevibacterium linens*, could be used as possible symbionts to prevent *Leishmania* transmission by vectors. A summary of genetic control approaches has been summarized in Table 2. Laboratory/field based information of different sand fly control approaches and their implications for future field applications are indicated in Table 3.

**Table 2:** The summary of genetic control approaches.

Technique	Description	Advantages	Limitations
Genetic modification	Involves altering the genetics of sand flies to produce sterile male insects or to employ transgenic approaches using advanced gene editing tools like CRISPR/Cas9. Microinjection of genetic constructs into early life stages, such as eggs, is a common method used for modification.	Potential to suppress vector populations. Utilizes existing gene drive techniques. Ability to create genetically modified organisms (GMOs) for vector control.	Limited research on the application of gene editing in sand flies. Requires detailed protocols for microinjection and addressing issues related to non-model organisms. Low survival rates in early trials.
CRISPR/Cas9 technique	A powerful genome editing tool that allows for the precise alteration of DNA in organisms. It has been used in several vector insects, but there are no reported attempts to apply it to sand flies. Studies have documented protocols for microinjection, which is essential for utilizing this system for sand fly control.	High efficiency of mutagenesis. Potential for generating specific modifications that can impact *Leishmania* transmission. Well studied in other vector species.	Challenges in gene editing and microinjection for non-model organisms. Need for successful protocols to achieve effective genetic modification.
Paratransgenesis	A technique that genetically modifies symbiotic microorganisms to express genes interfering with pathogens. The process includes isolating native symbionts from vector species, modifying them, and reintroducing them to interrupt the pathogen’s life cycle. Common microorganisms used include bacteria and occasionally fungi or viruses. This technique can also involve using endosymbiotic bacteria like *Wolbachia* as a biocontrol candidate.	Can provide a novel method to control vector-borne diseases. Potential for long-term solutions as modified symbionts can persist in the insect population.	Requires availability of culturable microorganisms. Must ensure the safety and non-pathogenicity of modified microbes. Challenges in delivery mechanisms and maintaining the fitness of symbionts.

**Table 3:** Summary of laboratory and field-based data for various sand fly control methods, with an analysis of their implications for future field applications.

Technique	Study type	Target vector/s	Outcomes	Impact on disease prevention	References
Indoor residual spraying (IRS)	Field studies	*Phlebotomus* species	IRS with pyrethroids (e.g., deltamethrin, alpha-cypermethrin) has shown significant sand fly suppression (>90%)	Effective in reducing sand fly populations in multiple regions (Bangladesh, India, Nepal); decrease disease transmission	[13,15,16,18,19]
Insecticide-treated durable wall lining (DWL)	Field studies	*Phlebotomus* species	Significant reduction in sand fly populations using polyethylene nets treated with insecticides	Alternative to IRS; effective in reducing local sand fly populations but limited by high cost and disposal issues	[5,13,22]
Insecticide-treated nets (ITNs)	Field trials	*Phlebotomus* species	Varying effectiveness in reducing sand fly populations using pyrethroids or deltamethrin and effective in lowering leishmaniasis burden	Effective in reducing disease burden, but issues with insecticide resistance, logistics, and short residual efficacy	[5,9,24,25]
Long lasting insecticide-treated nets (LLINs)	Field trials	*Phlebotomus* species	Significant reduction in the incidence of new cases and proven prolonged efficacy (9%–78% reduction in vector density) over nearly 2 years	Consistently effective in reducing vector populations over longer periods, although resistance and logistical challenges remain	[15,24]
Combined control measures (IRS + LLINs+ KOTAB)	Field study	*Phlebotomus* species	Significant reduction in sand fly populations (16%–86%) with combined interventions	Promising results when integrated with other vector control methods like IRS, enhancing overall disease control	[15]
Space spraying/fogging	Field study/pilot study	*Phlebotomus* species, *Lutzomyia* species	Mixed results in the control of sand flies; effective in some ecological settings with thermal fogging	Limited residual activity; generally ineffective in habitats with poor coverage (e.g., caves, crevices)	[12,28–30]
Impregnated dog collars	Field study/randomized controlled trial	*Lutzomyia* and *Phlebotomus* species	Effective reduction in sand fly contact with canine reservoirs using deltamethrin-treated collars	Proven to reduce infection risk in dogs and significantly lower the incidence of infantile visceral leishmaniasis	[34–36]
Entomopathogenic nematodes (EPNs)	Laboratory study/pilot study	*Phlebotomus* species, *Lutzomyia* species	Successful mortality in sand fly larvae (42%–94%) by EPNs from Steinernematidae and Heterorhabditidae families	Effective as a biocontrol agent, rapid mortality (24–48 h), potential for large scale use in soil habitats	[10,43,46]
Entomopathogenic protists	Laboratory study/experimental studies	*Phlebotomus* species, *Lutzomyia* species	Gregarine species like *Psychodiella chagasi*, *Ps. sergenti*, and *Ps. tobbi* infect sand flies, reducing their longevity and reproduction	Reduced vector longevity and reproduction in sand flies, showing potential for biocontrol; further studies needed	[10,47,48]
Mites	Laboratory studies	*Phlebotomus* species, *Lutzomyia* species	Significant reduction in sand fly larvae, pupae, and adult populations by parasitic and phoretic mites	Mites potentially offer a sustainable alternative to chemical insecticides, but field trials are needed for confirmation	[10,40,51,81]
Spiders (natural predation)	Field study/observational study	*Phlebotomus* species	Spiders observed preying on adult sand flies, including blood-fed females	Potential for reducing sand fly populations and reproductive capacity, more field studies are needed	[40]
Entomopathogenic fungi (EPF)	Laboratory studies/field studies	*Phlebotomus* species	EPF, such as *Beauveria bassiana* and *Metarhizium anisopliae*, effectively reduced sand fly populations. Both species exhibit varied effectiveness depending on sand fly species and growth stages	EPF has potential as an alternative vector control strategy for leishmaniasis, but field application effectiveness depends on delivery mechanisms and environmental factors	[55,57,59,60]
*Wolbachia*	Laboratory study	*P. papatasi, Lu. longipalpis*	Successful in vitro mutagenesis of sand fly vectors, producing increased susceptibility to *Leishmania*	Potential to reduce sand fly populations by altering reproductive capabilities or enhancing disease resistance	[63,64]
Sterile insect technique (SIT)	Conceptual & experimental research	*P. papatasi, Lu. longipalpis*	Release of sterile males to reduce mating success and control populations	Potential for long-term suppression of sand fly populations, reducing *Leishmania* transmission	[65,66]
CRISPR/Cas9 gene editing	Laboratory studies/experimental trial	*P. papatasi, Lu. longipalpis*	Development of gene editing protocols for sand fly microinjection and gene alteration successful in vitro mutagenesis of sand flies to increase susceptibility to *Leishmania* infection	Provides precise genetic modifications to reduce sand fly vector capacity for *Leishmania* Could potentially enhance vector control by making sand flies more susceptible to diseases	[69,70]
*Wolbachia* and SIT combination	Conceptual study	*P. papatasi, Lu. longipalpis*	Investigating the potential for using both *Wolbachia* and SIT together	Enhanced suppression of sand fly populations and possibly improved disease prevention	[71]
Paratransgenesis (bacterial symbionts)	Laboratory/field study	*Phlebotomus* species	Successful use of genetically modified bacteria (e.g., *Bacillus subtilis*) to disrupt *Leishmania* development in sand fly midgut	It can potentially reduce *Leishmania* transmission by altering the vector microbiome, but the delivery system still needs optimization	[73,78,80]

## Discussion

Biological and genetic control strategies exist as the most sustainable approaches for controlling disease vectors and minimizing case incidences that they cause. Natural parasites, predators, pathogens, and novel genetically modified control strategies taking attention over existing traditional control measures. The current review has explored some interesting biocontrol approaches, including entomopathogens, paratransgenic microbes as well as genetic approaches, which are used/can be used as practical application methods to control leishmaniasis vectors in the places where this disease is endemic and play significant social and economic burden to the society.

The sustainability and ecological safety of biological control exist as one of the key advantages compared to chemical insecticides [82]. Many of the discussed entomopathogenic organisms, including nematodes and mites, cause less harm to non-target organisms and thus may ensure public health safety. However, due to dependency on several external criteria like environmental factors [83], these may require repeated application when targeting large scale control interventions and improper application of these methods could make them outcompete natives for resources, thus reducing natural populations. Similar to chemical control methods, biological control agents may also face challenges related to resistance. For example, around 27 species of insects have been reported as resistant to *Bacillus thuringiensis* toxins [84]. Continuous pressure from biocontrol approaches may lead to the selection of resistant sand fly populations, ultimately undermining the effectiveness of these control methods.

Genetic control methods offer promising alternatives to traditional insecticides by targeting sand fly populations at the genetic level. For example, the SIT involves releasing sterilized male sand flies to compete with wild males, leading to population declines. While SIT has proven effective in mosquitoes [66], sand flies present unique challenges, such as lower population densities and breeding behavior differences, which require tailored approaches for SIT to be practical in field settings. Gene editing technologies, particularly CRISPR/Cas9, present another powerful tool, allowing precise modifications to sand fly genes associated with reproduction or pathogen transmission. Recent advances in CRISPR/Cas9 applications for other vector insects highlight the potential for similar changes in sand flies. However, the need for refined methods to achieve stable gene expression and successful microinjections remains a key focus for future research.

Additionally, gene drive systems engineered to spread a genetic trait through a population could be used to disseminate genes that reduce sand fly longevity or capacity for disease transmission, as seen in promising mosquito studies. *Wolbachia*-based approaches also offer potential; while not yet fully explored in sand flies, *Wolbachia* bacteria have effectively reduced vector populations and transmission rates in mosquito species [85]. Paratransgenesis, involving genetically modified symbiotic bacteria that inhibit pathogen development within the sand fly host, shows promise as an indirect genetic approach.

The main plus point of genetic control is the potential use of vector population suppression with less environmental disturbance and other non-targets. If the practical application of such a genetic control approach succeeds, it might reduce vector population over a long period without repeated application. The sterile male insect technique has many advantages like high specificity. Due to its principle that uses mate seeking behavior of insects, there is no need to rely on human interventions upon releasing them to the wild makes it a more conventional strategy [66]. Still, the sterilization process may reduce the longevity and mating potential of males [86]. Release of insects carrying a dominant lethal (RIDL) is an advanced form of SIT, which is effective using action and cost [86].

The gut and salivary proteins of sand flies are known to have an ambivalent influence on parasite development. Upon ingesting the infected blood meal, *Leishmania* parasites must contend with digestive enzymes that threaten their survival. Proteins in the sand fly mid gut help parasites to resist these enzymatic attacks. Certain developmental stages of *Leishmania*, such as procyclic promastigotes, show increased resistance to midgut proteases, ensuring their survival in the early phases of infection [87]. On the other hand, sand fly protein activity might adversely affect parasite development. A study by Pruzinova and team [88] suggested that parasites of leishmaniasis (*L. major* and *L. donovani*) could be destroyed due to the toxins produced during blood meal digestion.

Moreover, sand flies’ salivary proteins play a vital role in modulating the host’s immune response to facilitate *Leishmania* infection. During a bite, sand flies introduce saliva and *Leishmania* promastigotes into the host’s skin. The pharmacologically active components of saliva counteract the host’s hemostatic response and immune defenses. These proteins prevent blood clotting, enabling efficient feeding while suppressing local immune responses. Repeated exposure to sand fly saliva is also known to elicit specific antibodies, indicating that the immune system recognizes salivary proteins as foreign.

The dual role of sand fly proteins is both to facilitate *Leishmania* development and modulate host immunity, which presents opportunities for innovative control strategies. One promising approach is the development of vaccines targeting sand fly saliva. Immunization with salivary proteins has shown the potential to block the parasite-promoting effects of saliva, thereby limiting infection. Such vaccines could prevent the immunosuppressive environment created by sand fly bites, offering protection against *Leishmania* even before infection occurs [89].

Studying vector populations and their prevalence requires a combination of field surveys, laboratory analysis, and advanced statistical modeling. Field surveys are essential for collecting data on the abundance, distribution, and behavior of vectors in different environments. These surveys typically involve various sampling methods, such as light traps, sticky traps, baited traps, aspirators, and larval collection, to capture both adult and larval stages of vectors [90]. These methods help determine the density of vector populations and their geographical spread. In addition, entomological indices, such as the vectorial capacity and infection rate, are often calculated to assess the potential of vector populations in transmitting disease. Molecular techniques like polymerase chain reaction are increasingly used for identifying the species composition of vector populations, detecting infection rates, and determining insecticide resistance patterns.

In some cases, mark-recapture methods are employed to study the longevity, mobility, and survival rates of vectors in the wild [91]. Another valuable tool is geographic information systems, which help map vector distribution and model environmental factors influencing vector populations. Mathematical and statistical models, such as spatial analysis and forecasting models, predict vector prevalence and disease transmission under different ecological and intervention scenarios. These models are often validated with empirical data from field studies to improve their accuracy and reliability. By combining these various methodologies, researchers can understand vector dynamics comprehensively, which is crucial for effective disease control and prevention strategies.

IVM programs, which combine multiple vector control strategies, can potentially reduce the ecological impact compared to reliance on single methods. Incorporating chemical and biological control measures with personal protection and environmental management would be an excellent phenomenon. When designing a control scheme for a target insect, several factors should be carefully considered to achieve objectives. For vector species, thorough consideration of environmental and ecological factors such as the availability of resting, breeding grounds, and natural predators is essential as these play a critical role in determining vector density within particular geographics. On the positive side, IVM approaches that include environmental management practices such as habitat modification and biological control can reduce the need for chemical interventions, leading to lower ecological contamination and less harm to non-target species. Local community engagement might play an important role in IVM programs. Local communities may be hesitant to accept these strategies due to unawareness and concerns about the potential risks associated with introducing non-native species or pathogens into the environment [92]. Effective communication and education about the benefits and safety of biological and genetic control are essential for gaining public support. Engaging local communities in decision-making can help build trust and promote acceptance of biological control measures. Therefore, it is better to incorporate their volunteer participation in the control plan process, and providing awareness regarding these control strategies could be more advantageous to minimize potential challenges associated with implementing these strategies in practice. While IVM programs can provide sustainable and effective solutions for vector control, they require careful planning, monitoring, and adaptive management to minimize unintended ecological impacts. Regular ecological assessments and including environmental safeguards in IVM strategies will ensure that these programs support public health goals and environmental sustainability.

Yet, the long-term sustainability of these genetic approaches to *Leishmania* vector control is still uncertain, and evolutionary modifications within population genes could affect deficiency over time. Several challenges, like regulatory issues and community acceptance issues, hinder proper interventions of these methods. Therefore, field trials and multidisciplinary research are essential to address practical and ethical considerations, ultimately establishing genetic methods as sustainable tools for integrated sand fly management.

### Recommendation and way forward

Genetic control approaches, such as using GMOs and SITs, have shown significant promise in providing long-lasting control over vector populations. Studies indicate that these methods can reduce disease transmission over extended periods, with some approaches even demonstrating the potential to eliminate the need for chemical insecticides. Furthermore, these approaches reduce the environmental burden associated with chemical pesticides, as they specifically target the vector species and minimize non-target effects, making them a sustainable option for IVM. However, future research is essential to overcome the challenges allied with the genetic control options and assess their ecological impacts to ensure their safe and effective implementation in IVM programs.

Implementing control measures by addressing challenges and limitations associated with vector-borne disease control is advantageous. An effective delivery mechanism should be used to achieve optimal outcomes of these control strategies. The employment of collaborative partnerships among professionals in genetics, ecology, entomology, and public health would be a great way to address challenges associated with current control methods and design future control strategies.

It is essential to consider the proven biological and gene drive control measures successfully employed for mosquito control, including SIT and the release of genetically modified insects that should be tested as a potential control measure against sand flies. The priority should be given to an autonomous control measure. This could be achieved by incorporating various techniques and methodologies for a well-planned practice. As the disease leishmaniasis is well established in geographics with poor health and life resources, the most significant factor is that these integrated programs should be affordable and cost-effective for almost all countries with high disease burden. It is better to consider these factors before designing and implementing a control strategy to mitigate the leishmaniasis disease burden.

A well-planned integration among biological, chemical, genetic, and socio-economic opinions for sand fly control is crucial for applying advancing strategies. Continuous lab and field-based research, collaboration among experts in this field, and adapting control measures would be great approaches to addressing emerging challenges and constraints associated with the current control system. To ensure sustainable and successful implementation, integrated programs should prioritize affordability and accessibility, especially for low- and middle-income populations with high disease burdens. By doing so, health officials can implement sustainable integrated solutions to overcome the leishmaniasis disease burden in endemic countries and ultimately enhance public health outcomes worldwide.

### Key learning points

- Traditional insecticide-based methods for controlling sand fly populations face limitations, including the development of resistance and environmental concerns.
- Genetic control methods, such as SIT, RIDL, gene drive systems, and paratransgenesis, offer promising alternatives for managing sand fly populations.
- Gene editing in sand flies using CRISPR/Cas9 shows promise in reducing vector competence for leishmaniasis. Studies have demonstrated potential applications in *Phlebotomus papatasi* and *Lutzomyia longipalpis*.
- Combining multiple control methods, such as thermal fogging, gene editing, *Wolbachia* infection, and gut microbiome alterations, may provide the most effective approach to managing sand fly populations and controlling leishmaniasis.
- Each control method presents unique challenges, including technical limitations, ecological impacts, and potential resistance development. Further research is necessary to optimize these methods, evaluate their long-term effectiveness, and assess their effects on non-target organisms.

### Five key papers

1. Balaska S, Fotakis EA, Chaskopoulou A, Vontas J. Chemical control and insecticide resistance status of sand fly vectors worldwide. Guizani I, editor. PLOS Neglected Tropical Diseases. 2021 Aug 12;15(8):e0009586.
2. Slavica Vaselek. The role of protists, nematodes and mites as natural control agents of sandfly populations. Frontiers in Tropical Diseases. 2024 Mar 28. https://doi.org/10.3389/fitd.2024.1369007.
3. Dinesh DS, Kumar V, Kesari S, Das P. Mites and spiders act as biological control agent to sand flies. Asian Pacific Journal of Tropical Disease. 2014;4:S463–6.
4. Huda MM, Ghosh D, Alim A, Almahmud M, Olliaro PL, Matlashewski G, et al. Intervention packages for early visceral Leishmaniasis case detection and sandfly control in Bangladesh: a comparative analysis. The American Journal of Tropical Medicine and Hygiene [Internet]. 2019 Nov 19 [cited 2023 Apr 17];100(1):97–107. Available from: https://www.ajtmh.org/view/journals/tpmd/100/1/article-p97.xml
5. Wilson AL, Courtenay O, Kelly-Hope LA, Scott TW, Takken W, Torr SJ, et al. The importance of vector control for the control and elimination of vector-borne diseases. Barrera R, editor. PLOS Neglected Tropical Diseases. 2020 Jan 16;14(1):e0007831.
